
JOURNAL INFORMATION
==============================
NLM Title Abbreviation: Wirtschaftsdienst
Journal ID: Wirtschaftsdienst
NLM Journal ID: 101086612

Wirtschaftsdienst (Hamburg, Germany : 1949)

ISSN: 0043-6275

ARTICLE INFORMATION
==============================
PMCID: PMC7765698
PMID: 33390621
DOI: 10.1007/s10273-020-2797-x
Article ID: 2797
Article version: 1
Subjects: Zeitgespräch

Der amerikanische Traum ist ausgeträumt

Hüther Michael huether@iwkoeln.de

grid.473597.b 0000 0000 9854 4639 Institut der deutschen Wirtschaft Köln e.V., Konrad-Adenauer-Ufer 21, 50668 Köln, Deutschland
Electronic publication date: 2020 Dec 26
Print publication date: 2020
Volume: 100
Issue: 12
First page: 918
Last page: 923
Copyright: © Der/die Autor(en) 2020
License: Dieser Artikel wird unter der Creative Commons Namensnennung 4.0 International Lizenz (https://creativecommons.org/licenses/by/4.0/deed.de) veröffentlicht.
Open Access wird durch die ZBW — Leibniz-Informationszentrum Wirtschaft gefördert.
License URL: https://creativecommons.org/licenses/by/4.0/

issue-copyright-statement: © The Author(s) 2020

==============================

Die wirtschaftliche Lage und deren Perspektiven in den USA werden in besonderer Weise von den politischen Strukturen reflektiert. Denn die ökonomische Segregation in den vergangenen Jahrzehnten, die sich in einer historisch niedrigen Mobilität der Arbeitskräfte manifestiert, führt zu einer sich weiter ausprägenden Spaltung des Landes hinsichtlich Lebensführung, Qualifikation, wirtschaftlicher Basis und sozialen Perspektiven. Abbildung 1 zeigt, dass die Mobilität zwischen den Countys (Landkreisen) in den USA zwischen 1950 und 1992 nie unter 6 % gesunken ist, seit 2007 hat dieser Anteil hingegen 3,9 % nie überschritten. Der erste steile Rückgang trat zwischen 1990 und 1995 auf, der zweite nach 2005. Dies spiegelt sich im Rückgang der Mobilitätsrate innerhalb der Landkreise wider, die von über 13 % in den 1950er Jahren auf unter 6 % zurückging und sich somit mehr als halbiert hat.

Die Mobilität der Beschäftigten war ein wichtiges Merkmal des amerikanischen Wohlstandsversprechens (Austin et al., 2018): Man kann es schaffen, zur Not an anderer Stelle. Die Menschen wandern zu den Jobs. Dass dieses Versprechen nicht mehr wie früher trägt, das unterstreicht auch die generative Betrachtung. So zeigen Chetty et al. (2017) für die USA, dass der Anteil der Kinder, die ein höheres Familien- oder Arbeitseinkommen erzielen als ihre Eltern, dramatisch zurückgegangen ist (vgl. Abbildung 2). Lag in der Geburtskohorte von 1940 die Quote bei beiden Einkommensarten noch über 90 % und in der 1950er Kohorte bei 80 %, so lag sie in der jüngsten Geburtskohorte von 1984 bei 50 % und weniger. Dabei ist der Rückgang der absoluten Mobilität bei den individuellen Einkommen von Männern stärker als bei den Familieneinkommen insgesamt, was auf gestiegene Erwerbsbeteiligung und Einkommen von Frauen zurückzuführen ist. Der amerikanische Traum, man könne einfach zu den Jobs wandern, ist ausgeträumt; zumal insgesamt die Erwerbstätigenquote seit 2000 um 3,6 Prozentpunkte auf 60,8 % gesunken ist (OECD, 2020).

Abbildung 1
**Inländische Mobilität in den USA**
Anteil an der Gesamtbevölkerung

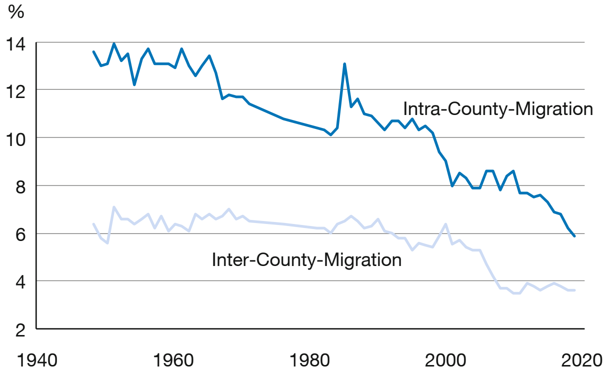

Anmerkungen: Möglicher Strukturbruch im Jahr 2006 aufgrund von Veränderungen beim Imputationsverfahren des Current Population Surveys, keine Daten für 1972 bis 1975 und 1977 bis 1980.

Quelle: U.S. Census Bureau (2019).

Abbildung 2
**Generative Einkommensmobilität in den USA**
Geburtskohorten gegenüber den Eltern; Anteil der Kinder, die ein höheres Einkommen erzielen als ihre Eltern

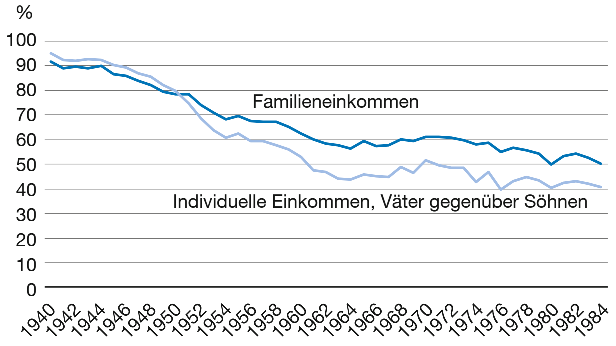

Quelle: Chetty et al. (2017).

Dieser Verlust an Einkommensaufstieg über die Generationen, der ebenso zum amerikanischen Wohlstandsversprechen gehörte, spiegelt sich auch in der Verteilung von Einkommen und Vermögen (Peterson Institute, 2020): Nirgends in der entwickelten Welt hat sich in den vergangenen Jahrzehnten die Ungleichheit der Markteinkommen – gemessen am Gini-Koeffizienten – so verschärft wie in den USA, und das bei dem höchsten Niveau im betrachteten Ausgangszeitpunkt 1985. Die 10 % einkommensstärksten Haushalte in den USA vereinigen 29 % aller Einkommen, die 10 % vermögensstärksten Haushalte 79 % des Gesamtvermögens (in Dänemark liegen die Anteile bei 22 % und 64 %, in Deutschland bei 23 % und 60 %) (Peterson Institute, 2020).

War in früheren Jahrzehnten die Wählergunst für die Republikaner und Demokraten nicht per se geografisch in regionalen Clustern von Staaten festgelegt, so hat sich dies seit den 1990er Jahren geändert. Seitdem sind die Staaten an der Westküste und an der nördlichen Ostküste sicher der demokratischen Partei zuzurechnen, die sogenannten flyover states der republikanischen Partei. Im Rust Belt gibt es viele Swing States, was dem historischen Wechsel beider Parteien als Interessenvertretung der industriell arbeitenden Mittelschicht entspricht. Zugespitzt gilt, dass die Demokraten die innovativen Zentren (Silicon Valley, Biotech Hub um Boston, Financial Center New York) gewinnen können, während die Republikaner in den traditionellen Wirtschaftsräumen verankert sind. Mit dieser Differenzierung verbinden sich unterschiedliche Einkommensniveaus (vgl. Hüther und Goecke, 2016; Packer, 2014). Hillary Clinton hat es im Rückblick auf die Präsidentschaftswahl 2016 so formuliert: „So I won the places that are optimistic, diverse, dynamic, moving forward. And his whole campaign, ‚Make America Great Again', was looking backwards." (Clinton, 2018).

Das signalisiert einen historischen Wandlungsprozess, denn die Demokratische Partei war traditionell „die Partei der Arbeiterschaft gewesen. Aber zu Beginn der 1970er Jahre, in einer Zeit, in der die Republikanische Partei die männliche weiße Arbeiterschaft umwarb, vor allem die Männer, die ihre Arbeitsplätze in der Industrie verloren hatten, begann die Demokratische Partei, die Arbeiterschaft aufzugeben, in erster Linie weiße Männer, und zwar zugunsten einer Koalition von Frauen, Minderheiten und einem Personenkreis, der mittlerweile als ‚knowledge workers' bezeichnet wurde…" (Lepore, 2019, 843). Darauf hat die Demokratische Partei bis heute keine wirksame Antwort gefunden, die Republikaner sehen für sich überhaupt keine Herausforderung in der skizzierten Verschiebung.

Politisch hat diese Differenzierung der Parteistammwählerschaft zu einer programmatischen Homogenisierung beider Parteien seit den 1970er Jahren geführt, die über die Jahre eine immer wirkmächtigere Formung gefunden hat (Lepore, 2019, 788 ff.). Während die Demokraten in Nachfolge der liberalen Erfolge der 1960er Jahre und vor allem Johnsons Great Society-Programms sich immer weiter an der Identitätspolitik ausgerichtet haben, wandte sich die Grand Old Party seit den frühen 1970er Jahren von zuvor vertretenen liberalen Positionen – zu Gleichberechtigung, Abtreibung, Homosexualität, Einwanderung, Rassengleichstellung, Waffenbesitz – ab. „Konservative begründeten ihren Machtanspruch mit dem Scheitern des Liberalismus, das in den 1960er Jahren begann, als

Abbildung 3

**Beschäftigte im Verarbeitenden Gewerbe der USA**

Vollzeitäquivalente (VZÄ)

der Begriff der Identität den Begriff der Gleichheit ersetzte“ (Lepore, 2019, 960). So verschärft die politische Spaltung die sie erzeugende ökonomische Segregation.

## Herausforderungen für die Biden-Administration

Die ökonomischen und politischen Strukturen sind für die Analyse der wirtschaftspolitischen Perspektiven so bedeutsam, weil sie in ihrer Verhärtung den Spielraum der neuen Administration definieren. Entsprechend finden sich von Joe Biden programmatische Hinweise aus der Wahlkampagne, die auf die Stärkung des industriellen Sektors und verbesserte Bedingungen für traditionelle Mittelklasse-Haushalte zielen (Biden, 2020). Im Rückblick auf das vergangene Jahrzehnt stellt man indes fest, dass seit der Finanzkrise der Abbau von Arbeitsplätzen im Verarbeitenden Gewerbe allenfalls gestoppt werden konnte (vgl. Abbildung 3). Der Anteil an allen Erwerbstätigen liegt mit unter 10 % nur halb so hoch wie in Deutschland, zugleich erweist sich die Einkommensperspektive in diesem Sektor als deutlich schwächer als hierzulande.

Dies entspricht auch dem Anteilsverlust des Verarbeitenden Gewerbes an der Bruttowertschöpfung in den USA, der verglichen mit Deutschland sehr viel stärker war; mit ebenfalls rund 10 % erreicht er nur die Hälfte des Anteils hierzulande (The World Bank, 2020). Eine wesentliche Erklärung für den Unterschied liegt in der unterschiedlich ausgeprägten Flexibilität, auf günstigere Importe vor allem aus China reagieren zu können. Für die USA zeigt sich, „that the increase in U.S. imports from China, which accelerated after 2000, was a major force behind recent reductions in U.S. manufacturing employment and that, through input-output linkages and other general equilibrium effects, it appears to have significantly suppressed overall U.S. job growth. […] Our central estimates suggest net job losses of 2.0 to 2.4 million stemming from the rise in import competition from China over the period 1999 to 2011“ (Acemoglu et al., 2016). Für Deutschland lassen sich zwar ebenfalls Jobverluste in jenen Sektoren ermitteln, die der chinesischen Importkonkurrenz ausgesetzt sind, aber insgesamt konnte die Exportorientierung der deutschen Industrie Wertschöpfung und Jobs neu entwickeln, sodass gilt, „that globalization did not speed up the manufacturing decline in Germany. It even retained those jobs in the economy“ (Dauth et al., 2017).

Die regionale Verdichtung wirtschaftlichen Wachstums in den USA ergibt sich auch daraus, dass die Wissensintensivierung vieler Berufe im Dienstleistungsbereich auf Netzwerk- und Skaleneffekten in Städten beruht. So war das jüngste Wirtschaftswachstum in den USA merklich auf die reichsten und größten Städte ausgerichtet. Eine Studie von Eckert et al. (2019/20) zeigt, welche Bedeutung dafür kompetenz- und informationsintensive Dienstleistungsbranchen und deren Konzentration an produktiven Standorten haben. Offenkundig finden sich Superstar-Unternehmen in Superstar-Städten und charakterisieren so das Wirtschaftswachstum in wissensbasierten Volkswirtschaften wie den USA, zugleich erklären sie die gewachsene regionale Ungleichheit zwischen Arbeitskräften und Regionen (vgl. Abbildung 4).

Diese Dienstleistungsbranchen weisen sowohl eine hohe Qualifikationsintensität auf als auch eine besondere internationale Handelbarkeit. Unternehmensberatung, Softwareentwicklung und Finanzdienstleistungen sind einige herausragende Beispiele. Seit 1980 erzielen die Arbeitskräfte in diesen Branchen im Vergleich zu allen anderen amerikanischen Arbeitskräften ein außerordentliches Lohnwachstum. Dieses schnelle Lohnwachstum hat jedoch nicht zu entsprechenden Beschäftigungszuwächsen für den Sektor geführt. Im Gegensatz dazu folgt das Beschäftigungswachstum dem Lohnwachstum in anderen Sektoren. Die talentiertesten Arbeiter*innen und Unternehmen für solche Dienstleistungen bedienen heute einen größeren Teil des Marktes, erhöhen ihr Einkommen und machen weniger talentierte Arbeiter*innen für handelbare Dienstleistungen überflüssig. Ein Blick auf die Beschäftigungszusammensetzung zeigt, dass der Anteil der Arbeitskräfte mit mindestens einem Hochschulabschluss, die in solchen Dienstleistungen arbeiten, schneller gewachsen ist als der Beschäftigungsanteil dieser Arbeitskräfte an der Gesamtwirtschaft.

Gegen diese Trends kann eine nationale Wirtschaftspolitik nur dann Wirkung entfalten, wenn sie auf eine regionalpolitische Strategie setzt, die neben Bildungsinfrastruktur – vor allem auch einer beruflichen Bildung – auch gezielt eine Ansiedlungspolitik betreibt, damit die Jobs zu den

Abbildung 4

**Transformation hochqualifizierter Jobs in den USA**

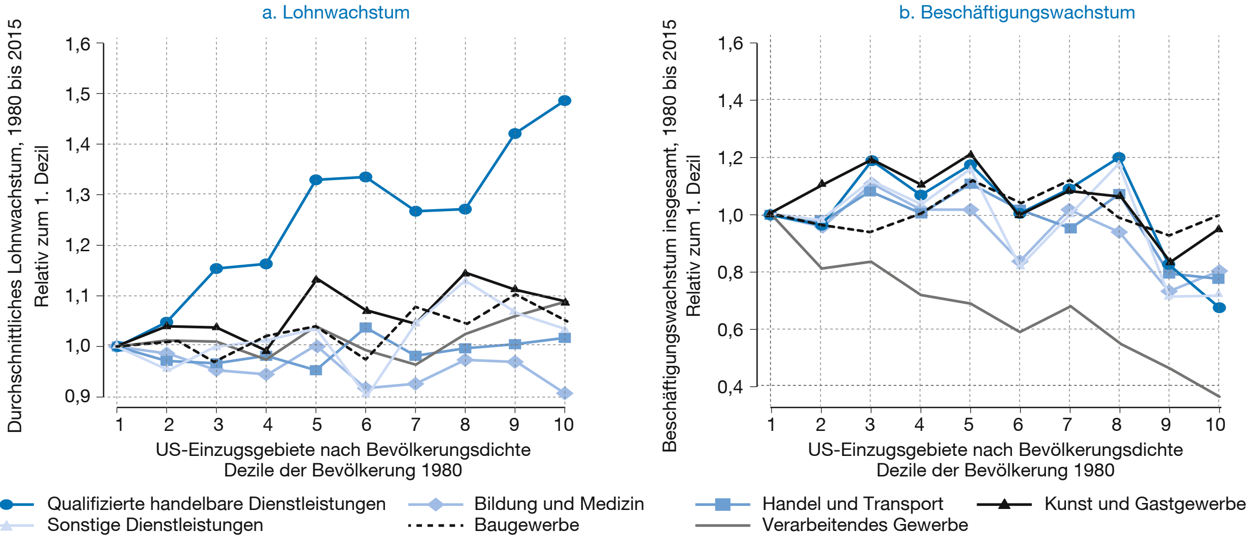

Quelle: Eckert et al. (2019/20).

Menschen kommen, wenn die Wanderung nicht mehr wie früher funktioniert. Während Regionalpolitik in Deutschland durch den Bund seit den frühen 1950er Jahren und in Europa im Rahmen der Kohäsionspolitik seit den 1960er Jahren betrieben wird, ist dies in den USA nicht der Fall. Damit sind regionale Pfadabhängigkeiten kaum zu durchbrechen. Die MIT-Studie zur Zukunft der Industrie in den USA hat den strategischen Nachteil in einem Mangel an Netzwerken und Clustern (Ökosystemen) verortet, was dazu führe, dass viele Industrieunternehmen isoliert und damit ohne Chance im internationalen Innovationswettbewerb seien (Berger, 2013). Auch hierauf liegt die Antwort in einer gezielten Entwicklung regionaler Verbünde. Deutschlands Vorteil liegt in der föderalen politischen Struktur begründet, welche die Industrialisierung seit dem 19. Jahrhundert durch die Schaffung von regionalen Clustern bis heute prägt und deshalb die Superstar-Städte in ihrer Bedeutung für die Einkommensentstehung kompensiert (Hüther, 2018).

## America First wirkte als America Alone

Es kann deshalb nicht verwundern, wenn weder die brachiale Außenwirtschaftspolitik noch die „America First“-Agenda von Donald Trump keine nachhaltigen Effekte entfalten konnte, der Vergleich mit den acht Jahren der Präsidentschaft von Obama ist deshalb ernüchternd (vgl. Abbildung 5). Das Leistungsbilanzdefizit gegenüber Deutschland bewegt sich auf stabilem Niveau (in Mrd. US-Dollar); gegenüber China, dem Hauptzielland der US-Zölle, lag das entsprechende Defizit 2019 mit 338 Mrd. US-$ auf gleichem Niveau wie 2016, nachdem es zwischenzeitlich auf mehr als 400 Mrd. US-$ angestiegen war (US Bureau of Economics Analysis, 2020b). Die USA verdienen mittlerweile nur noch 43 % der Erträge in ihrer Leistungsbilanz über Warenexporte, den Rest mit Dienstleistungen, durch Kapitaleinkünfte und Transfers. Die lange etablierten Trends und Muster der Spezialisierung im internationalen Handel lassen sich angesichts der damit verbundenen Globalisierung der Wertschöpfungsketten nicht so einfach aufbrechen, zumal dann nicht, wenn eine Regierung konsequent kontraproduktiv agiert: über höhere Staatsausgaben, geringere Steuern, geringere Ersparnis und höhere Investitionen.

Doch nicht nur außenwirtschaftlich hat Trump seine selbsterklärten Ziele nicht erreicht. Die Wachstumsdynamik folgt in etwa der unter Obama, der mit einer Vielzahl von konjunkturstimulierenden Maßnahmen das Erbe der Finanzkrise zu bereinigen versuchte. Die Zahl der Empfänger*innen von Lebensmittelgutscheinen ist zwar seit 2013 rückläufig, hat aber bislang das Niveau vor der Finanzkrise nicht wieder erreicht. Und dies, obwohl es bereits ab dem Jahr 2010 gelungen ist, die Arbeitslosenquote zu verringern; erst die COVID-19-Pandemie hat wieder zu einem abrupten und massiven Anstieg der Arbeitslosigkeit geführt. Beide Indikatoren zeigen ergänzend zu verteilungspolitischen Befunden (Peterson Institute, 2020), dass die Reduzierung der Arbeitslosigkeit nicht wie früher mit einer Verbesserung der Lebensverhältnisse verbunden war, zumal insgesamt die Erwerbstätigenquote aktuell deutlich unter dem Niveau des Jahres 2000 liegt.

Abbildung 5

**Was Trump (nicht) erreicht hat**

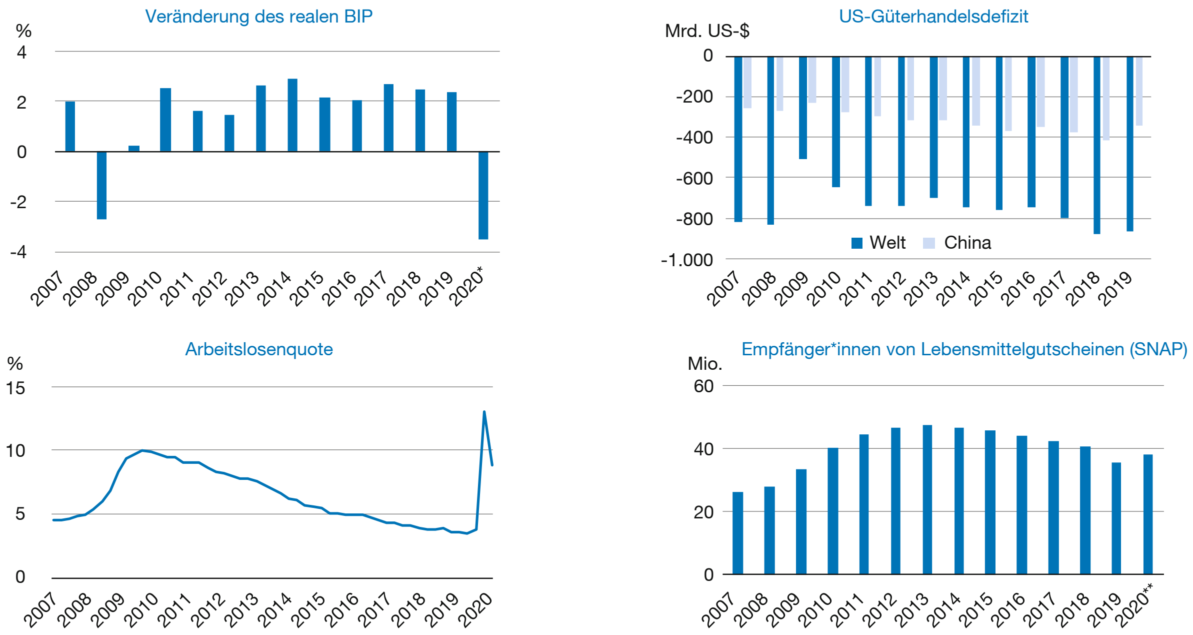

* Schätzung ab 2020; ** 2020 = Durchschnitt der Monate Oktober 2019 bis April 2020.

Vor diesem Erfahrungshintergrund ist kaum zu erwarten, dass der von Biden in Aussicht gestellte 700-Milliarden-Dollar-Plan – staatliche Ausgaben in Höhe von 400 Mrd. US-$ für US-Güter und Dienstleistungen und Investition von 300 Mrd. US-$ in Forschung und Entwicklung für Technologien wie Elektrofahrzeuge, leichte Materialien, 5G und künstliche Intelligenz – zu einem grundsätzlich anderen Ergebnis führen kann. Es dürfte vielmehr zu einer weiteren Profilierung der wissensbasierten Superstar-Communities kommen. Am ehesten sind regionale Effekte von den ebenfalls angekündigten 1,3 Billionen US-$ für Infrastruktur über ein Jahrzehnt zu erwarten (50 Mrd. US-$ im ersten Amtsjahr für die Instandsetzung von Straßen, Autobahnen und Brücken, 20 Mrd. US-$ für die ländliche Breitbandinfrastruktur, 400 Mrd. US-$ über zehn Jahre für Forschung und Innovation im Bereich saubere Energie, 5 Mrd. US-$ über fünf Jahre für die Batterietechnologie für Elektroautos und 10 Mrd. US-$ über zehn Jahre für Transitprojekte, die Gebiete mit hoher Armut versorgen).

Die Überwindung der ökonomischen Spaltung verlangt eine regionalpolitische Strategie, die sich den strukturell veränderten Realitäten stellt. Das aber setzt einen gesamtstaatlichen und überparteilichen Konsens voraus, der angesichts der politischen Spaltung nicht zu erwarten ist. Die dafür gebotene Erhöhung der direkten Steuern – man kann die USA als unterbesteuert bewerten – wird zudem ein unüberwindliches Hindernis im Senat bilden. Alles in allem ist die Wahrscheinlichkeit gering, dass Wirtschaftspolitik wirksam etwas an den sozialen Verwerfungen wird ändern können. Das wiederum verhärtet die schon harten politischen Fronten weiter. Dagegen ist eine liberale Außenwirtschaftspolitik nur vermittelbar, wenn sie die Parole „America first" in „Let's do better together" umwandelt, ohne die eigenen Interessen zu verleugnen, aber deren Durchsetzung gegen China und Russland zum gemeinsamen Anliegen des transatlantischen Westens macht. Hierfür gibt es mit Biden Hoffnung.

Prof. Dr. Michael Hüther ist Direktor des Instituts der deutschen Wirtschaft (IW) in Köln und Hochschullehrer an der European Business School in Oestrich-Winkel.


Literatur

Acemoglu, D., D. Autor, D. Dorn, G. H. Hanson und B. Price (2016), Import Competition and the Great US Employment Sag of the 2000s, NBER Working Paper, 20395, https://www.nber.org/system/files/wor-king_papers/w20395/w20395.pdf (25. November 2020).
Austin, B. A., E. L. Glaeser und L. Summers (2018), Jobs for the Heartland: Place-Based Policies in 21st Century America, NBER Working Paper, 24548, https://scholar.harvard.edu/files/glaeser/files/jobs_for_the_heartland_nberwp.pdf (25. November 2020).
Berger, S. (2013), Making in America. From Innovation to Market, The MIT Press.
Biden, J. (2020), The Biden Plan to Ensure the Future is „MADE IN ALL OF AMERICA” by all of America’s Workers, https://joebiden.com/made-in-america/ (25. November 2020).
Bureau of Economic Analysis (2020), U.S. International Transactions, https://apps.bea.gov/iTable/iTable.cfm?reqid=62&step=7&isuri=1&tablelist=30003&thetableflexibleareas=1&product=1&filter_—5=&tablelistsecondary=30009&filter_—4=114&filter_—3=25&filter_—2=0&filter_—1=2,3,4,5 (10. Dezember 2020).
Chetty R Grusky D Hell M Hendren N Manduca R Narang J The fading American dream: Trends in absolute income mobility since 1940 Science 2017 356 6336 398 406 10.1126/science.aal4617 28438988
Clinton, H. (2018), Speech at the India Today Conclave 2018 in Mumbai, https://www.businessinsider.com/hillary-clinton-says-trump-won-backwards-states-in-2016–2018-3?r=DE&IR=T (25. November 2020).
Dauth, W., S. Findeisen und J. Südekum (2017), Trade and Manufacturing Jobs in Germany, DICE Discussion Paper, Nr. 242, https://www.dice.hhu.de/fileadmin/redaktion/Fakultaeten/Wirtschaftswissen-schaftliche_Fakultaet/DICE/Discussion_Paper/242_Dauth_Findei-sen_Suedekum.pdf (25. November 2020).
Eckert, F., S. Ganapati und C. Walsh (2019/20), Skilled Scalable Services: The New Urban Bias in Economic Growth, Opportunity and Inclusive Growth Institute Working Papers, Nr. 25, Federal Reserve Bank of Minneapolis, https://www.minneapolisfed.org/institute/working-papers-institute/iwp25.pdf (25. November 2020).
Federal Reserve Bank of St. Louis (2020), Federal Reserve Economic Data, https://fred.stlouisfed.org (25. November 2020).
Hüther M Looking Back to the Future: Time Strata and Economic Analysis Journal of Contextual Economics — Schmollers Jahrbuch 2018 138 2 89 116 10.3790/schm.138.2.89
Hüther, M. und H. Goecke (2016), San Francisco Bay Area: 750 US-Dollar mehr für jeden US-Bürger, IW-Kurzbericht, Nr. 70, https://www.iwkoeln.de/fileadmin/publikationen/2016/305567/IW-Kurzbe-richt_70_2016_San_Francisco_Bay_Area.pdf (25. November 2020).
Lepore, J. (2019), Diese Wahrheiten. Geschichte der Vereinigten Staaten von Amerika, C.H. Beck.
OECD (2020), Labour Force Statistics, https://stats.oecd.org/Index.aspx?DataSetCode=lfs_sexage_i_r# (10. Dezember 2020). Packer, G. (2014), Die Abwicklung. Eine innere Geschichte des neuen Amerika, S. Fischer.
Peterson Institute for International Economics (2020), How to Fix Economic Inequality? An Overview of Policies for the United States and Other High-Income Economies, https://www.piie.com/sites/default/files/documents/how-to-fix-economic-inequality.pdf (25. November 2020).
The World Bank (2020), Manufacturing — Germany, United States, https://data.worldbank.org/indicator/NV.IND.MANF.ZS?locations=DE-US (10. Dezember 2020).
U.S. Bureau of Economic Analysis (2020a), U.S. International Trade in Goods by Area and Country, Seasonally Adjusted Detail.
U.S. Bureau of Economic Analysis (2020b), Table 1.3. U.S. International Transactions, Expanded Detail by Area and Country, https://apps.bea.gov/iTable/iTable.cfm?reqid=62&step=10&isuri=1&0=5&tablelist=30003&product=1 (10. Dezember 2020).
U.S. Census Bureau (2019), CPS Historical Migration/Geographic Mobility Tables, https://www.census.gov/data/tables/time-series/demo/geographic-mobility/historic.html (25. November 2020).
U.S. Department of Agriculture (2020), SNAP Data Tables, https://www.fns.usda.gov/pd/supplemental-nutrition-assistance-program-snap (25. November 2020).
